# Synergistic effect of cryptotanshinone and temozolomide treatment against human glioblastoma cells

**DOI:** 10.1038/s41598-023-48777-z

**Published:** 2023-12-09

**Authors:** Songxian Zhu, Jingjing Guo, Li Yu, Jun Liu, Jixiang Chen, Jinxin Xin, Yuqiang Zhang, Jie Luo, Chao Duan

**Affiliations:** 1 Brain Research Institute, Research Center of Neurological Diseases, Taihe Hospital, Hubei University of Medicine, 32 Renmin South Rd, Shiyan, 442000 Hubei China; 2 Department of Neurosurgery, Taihe Hospital, Hubei University of Medicine, 32 South Renmin Road, Shiyan, 442000 Hubei China; 3 Medical Services, Taihe Hospital, Hubei University of Medicine, 32 Renmin South Rd, Shiyan, 442000 Hubei China; 4 Hubei Key Laboratory of Wudang Local Chinese Medicine Research, Taihe Hospital of Shiyan, Hubei University of Medicine, Shiyan, 442000 Hubei China

**Keywords:** Cancer, Molecular biology, Molecular medicine

## Abstract

Glioblastoma multiforme (GBM) is a complex disease to treat owing to its profound chemoresistance. Therefore, we evaluated the combined effect and therapeutic efficacy of temozolomide (TMZ), a potent alkylating agent and the current gold standard therapy for GBM, and cryptotanshinone (CTS), which inhibits glioma cell proliferation in GBM cells. Using LN229 and U87-MG human GBM cells in a short-term stimulation in vitro model, the cytotoxic and anti-proliferative effects of single and combined treatment with 4 μM CTS and 200 μM TMZ were investigated. Furthermore, cell viability, DNA damage, apoptosis rate, and signal transducer and activator of transcription 3 (STAT3) protein were measured using cytotoxic assay, comet assay, flow cytometry, and western blotting analysis, respectively. The two drugs’ synergistic interaction was validated using the synergy score. We found that the anti-proliferative effects of combination therapy using the two drugs were greater than that of each agent used alone (CTS or TMZ). Western blot analysis indicated that treatment of GBM cells with CTS combined with TMZ more significantly decreased the expression of MGMT and STAT3, than that with TMZ alone. Combined treatment with CTS and TMZ might be an effective option to overcome the chemoresistance of GBM cells in a long-term treatment strategy.

## Introduction

Gliomas are the most common primary brain tumors, and over half of these are glioblastoma tumors, which are the most malignant^1^. A combination of surgery, radiotherapy, and/or chemotherapy is the primary strategy for glioblastoma multiforme (GBM) therapy^2^. Temozolomide (TMZ) is a first-choice alkylating agent and the gold standard therapy for GBM because of its ability to penetrate the blood–brain barrier, weaker adverse side effects, and higher effectiveness in extending the life span of patients^3,4^. The anti-tumor effect of TMZ depends on its ability to deliver a methyl group to purine bases of DNA (N7-guanine, N3-guanine, O6-guanine, and N3-adenine), which can induce cell cycle arrest at the G2/M phase and eventually lead to apoptosis^5–7^.

However, TMZ is ineffective against approximately 50% of primary or recurrent GBMs^8^. TMZ resistance is associated with the expression levels of DNA alkylating proteins and repair enzymes^9,10^. The endogenous enzyme O-6-methylguanine DNA methyltransferase (MGMT) binds to the alkyl compound on the sixth oxygen atom of DNA guanine and transfers the alkyl compound to the 145th cysteine active site of MGMT. Guanine, which causes DNA alkylation, is reduced, thus attenuating the killing effect of TMZ on tumor cells^11–13^. The absence of a mismatch repair (MMR) system is another critical mechanism that generates resistance in GBM^14,15^. Many other molecular mechanisms, including DNA repair systems, abnormal signaling pathways, autophagy, epigenetic modifications, microRNAs, and the production of extracellular vesicles, contributing to TMZ resistance have been revealed in recent years^16–19^. Although many of these mechanisms have been elucidated, the efficacy of TMZ has not improved. Thus, novel drugs and therapeutic protocols for the combined treatment of TMZ-resistant glioma cells are urgently required.

**Figure 1:**
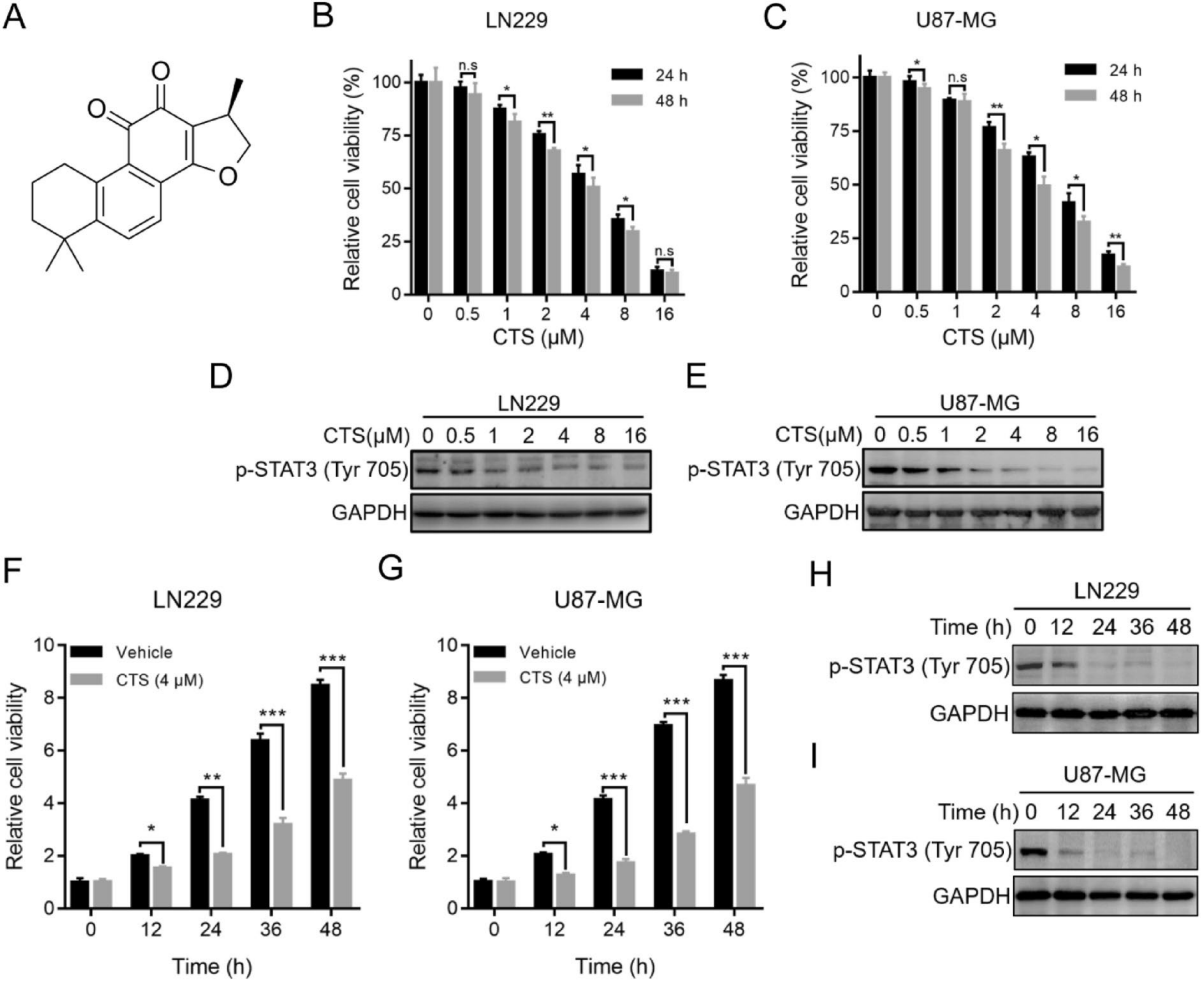
CTS treatment inhibits cell growth by suppressing STAT3 activity. (**A**) Chemical structure of CTS. (**B**,**C**) LN229 and U87-MG human GBM cells were treated with a series of concentrations of CTS for 24 or 48 h, and cell viability was determined with a CCK-8 assay. (**D**,**E**) Western blot analysis showing protein expression in LN229 and U87-MG cells using the indicated antibodies. (**F**,**G**) LN229 and U87-MG cells were treated with vehicle control or 4 μM CTS for different periods, and cell viability was determined with a CCK-8 assay. (**H**,**I**) LN229 and U87-MG cells were treated with vehicle control or 4 μM CTS for different periods, and protein expression was detected with the indicated antibodies (***P < 0.001, **P < 0.01, *P < 0.05).

A convenient and efficient method for discovering and developing new chemotherapy drugs is studying the anti-cancer properties of Chinese herbal medicines and their derivatives. Cryptotanshinone (CTS) (IUPAC name: (R)-1,2,6,7,8,9-hexahydro-1,6,6-trimethyl-phenanthro(1,2-b) furan-10,11-dione) (Fig. 1A) is a biologically active diterpenoid quinone compound extracted from fat-soluble constituents in the roots and rhizomes of *Radix* *Salvia* *miltiorrhiza*. CTS possesses various bioactivities, such as antibiotic^20,21^, anti-inflammatory^22^, anti-aging, and anti-tumor^23–25^ properties. The anti-cancer properties of CTS have drawn the attention of many researchers in recent years. Previous studies on the molecular mechanisms associated with CTS intervention have revealed that the JAK2/STAT3, PI3K/AKT, and cell cycle pathways are involved in the inhibitory and pro-apoptotic effects of this compound in different tumor cell lines^26^. CTS can inhibit the Tyr705 phosphorylation of signal transducer and activator of transcription 3 (STAT3) by binding to its SH2 domain, promoting the upregulated activity of the SHP-2 protein tyrosine phosphatase. It further blocks STAT3 dimerization and transcriptional activity after entering the nucleus, thus inhibiting the proliferation of tumor cells^27,28^. However, the inhibition of STAT3 phosphorylation is independent of its effect on JAK2 phosphorylation^24^. As an MGMT transcription factor, the activation and nucleation of Tyr705 in STAT3 are critical for DNA damage repair in tumor cells. Knocking down STAT3 can effectively reduce MGMT expression^29–32^. Therefore, screening compounds that directly target STAT3 and inhibit MGMT expression are of considerable significance for improving the efficacy of TMZ chemotherapy in glioma patients.

Currently, the anti-glioma effects of TMZ in combination with CTS have not been reported. Therefore, we aimed to address the synergistic sensitization effect of CTS and TMZ on GBM cells. The MGMT and STAT3 signaling pathways were evaluated to investigate the mechanism underlying the synergistic effect of CTS and TMZ through effective combination treatment.

## Results

### CTS blocks STAT3 activity and exhibits dose-dependent anti-proliferation effects

We first investigated the anti-proliferation effect of CTS, a putative STAT3 inhibitor, using cell viability assays on the LN229 and U87-MG human GBM cell lines. CTS (0–16 μM) was used to treat the two cell lines and inhibited growth in a dose-dependent manner; the concentration corresponding to a cell inhibition rate of 50% was 4 μM (Fig. 1B,C). Western blot analysis revealed that exposure of LN229 and U87-MG cells to CTS resulted in the suppression of STAT3 phosphorylation in a dose-dependent manner, suggesting that STAT3 activity was sufficiently blocked at a low dosage of CTS (Fig. 1D,E). As shown in Fig. 1F,G, the inhibitory effect of CTS treatment on the cell viability of both LN229 and U87-MG cells increased over time. Western blot analysis indicated that STAT3 phosphorylation was suppressed in a time-dependent manner and that STAT3 activity was sufficiently blocked at 24 h (Fig. 1H,I).

### Characterization of the anti-glioma cells effect of CTS and TMZ co-treatment

**Figure 2:**
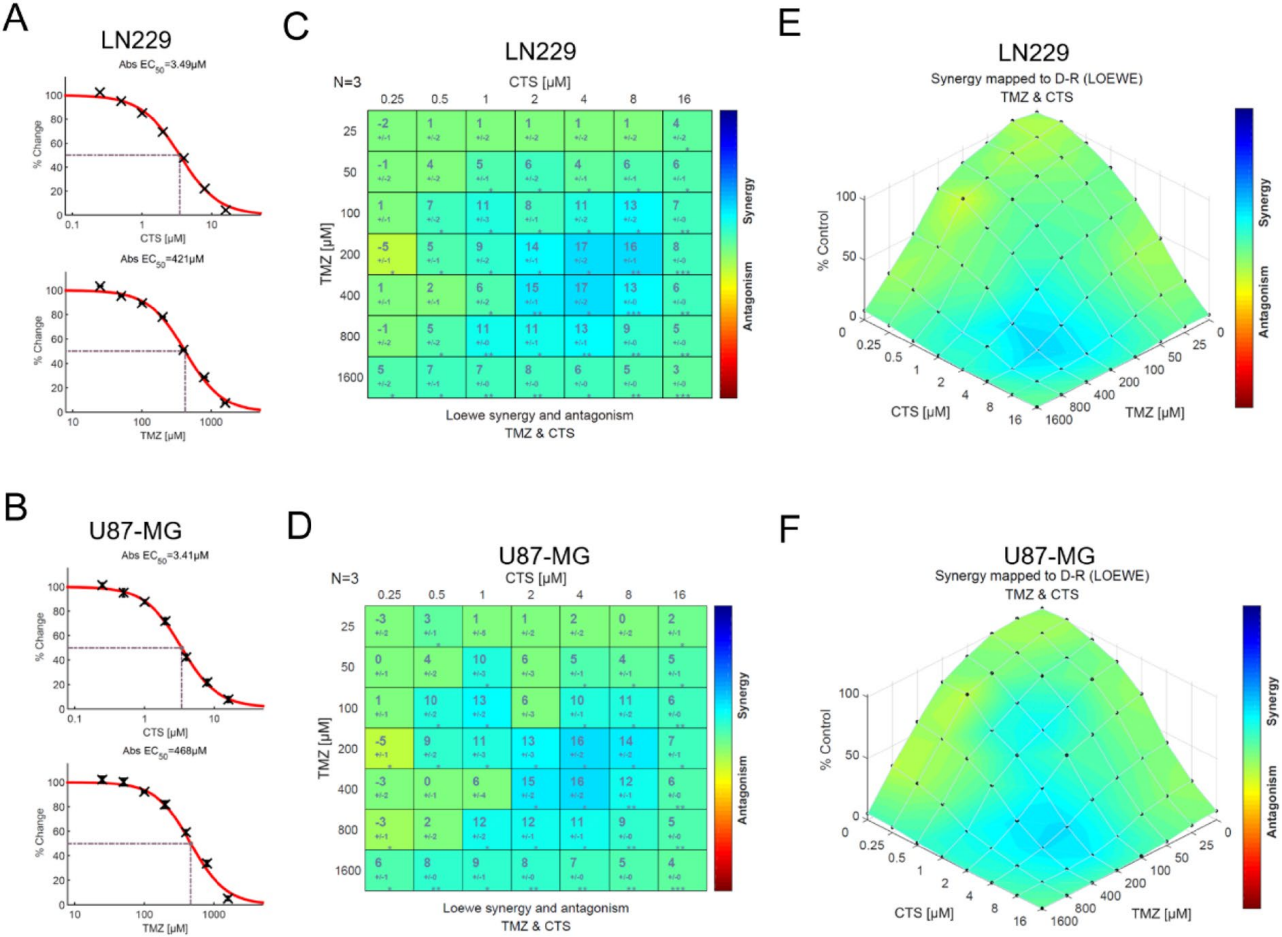
Characterization of the anti-glioma activity of a combination treatment of CTS and TMZ. (**A**,**B**) The IC50 value of CTS and TMZ as single-agent treatments of LN229 and U87-MG human GBM cells was verified with a CCK-8 assay. (**C**,**D**) Synergy plots generated by *Combenefit* depicting the interaction between CTS and TMZ. An analysis of interactions provided HSA and Bliss values (n = 3, technical replicates), indicating synergistic efficacy as calculated from expected and observed growth inhibition. HSA and Bliss values > 0 indicate synergistic effects. (**E**,**F**) Single and combinatorial titration of CTS and TMZ in a three-day growth assay of LN229 and U87-MG cell lines (***P < 0.001, **P < 0.01, *P < 0.05).

CTS has not yet been reported elsewhere for its combined effect with TMZ; therefore, it was selected for further investigation. The cell viability of 7-by-7 drug dose was detected by CCK-8 kit, and the inhibition rate of each concentration of drug on cell viability was calculated (the data is ordered with the first drug in columns and the second in rows). And then, synergy score of the two drugs combination was obtained by Combenifit software to evaluate the cell activity inhibition rate at different drug concentrations. As shown in Fig. 2A,B, LN229 and U87-MG cells were exposed to various 7-by-7 matrix concentrations of CTS (0.25–16 μM) and TMZ (25–1600 μM) for 72 h. The half-maximal inhibitory concentrations (IC50) of CTS against LN229 and U87-MG cells were 3.49 μM and 3.41 μM, respectively, and the IC50 of TMZ were 421 μM and 468 μM, respectively. In addition, classical synergy models, including the highest single agent (HSA) and Bliss reference models, were applied to determine HSA and Bliss values, which are readouts for synergistic inhibition and depict the difference between the expected inhibition and observed inhibition. Most HSA and Bliss values were above 0, indicating synergistic interactions between the two drugs (Fig. 2C,D). In addition, the expected versus observed dose–response surface plot of the 7-by-7 matrix viability data helped verify the dosage of the synergistic effect of the two agents against LN229 and U87-MG GBM cells (Fig. 2E,F).

### Combination treatment of GBM cells with CTS and TMZ suppresses proliferation

**Figure 3:**
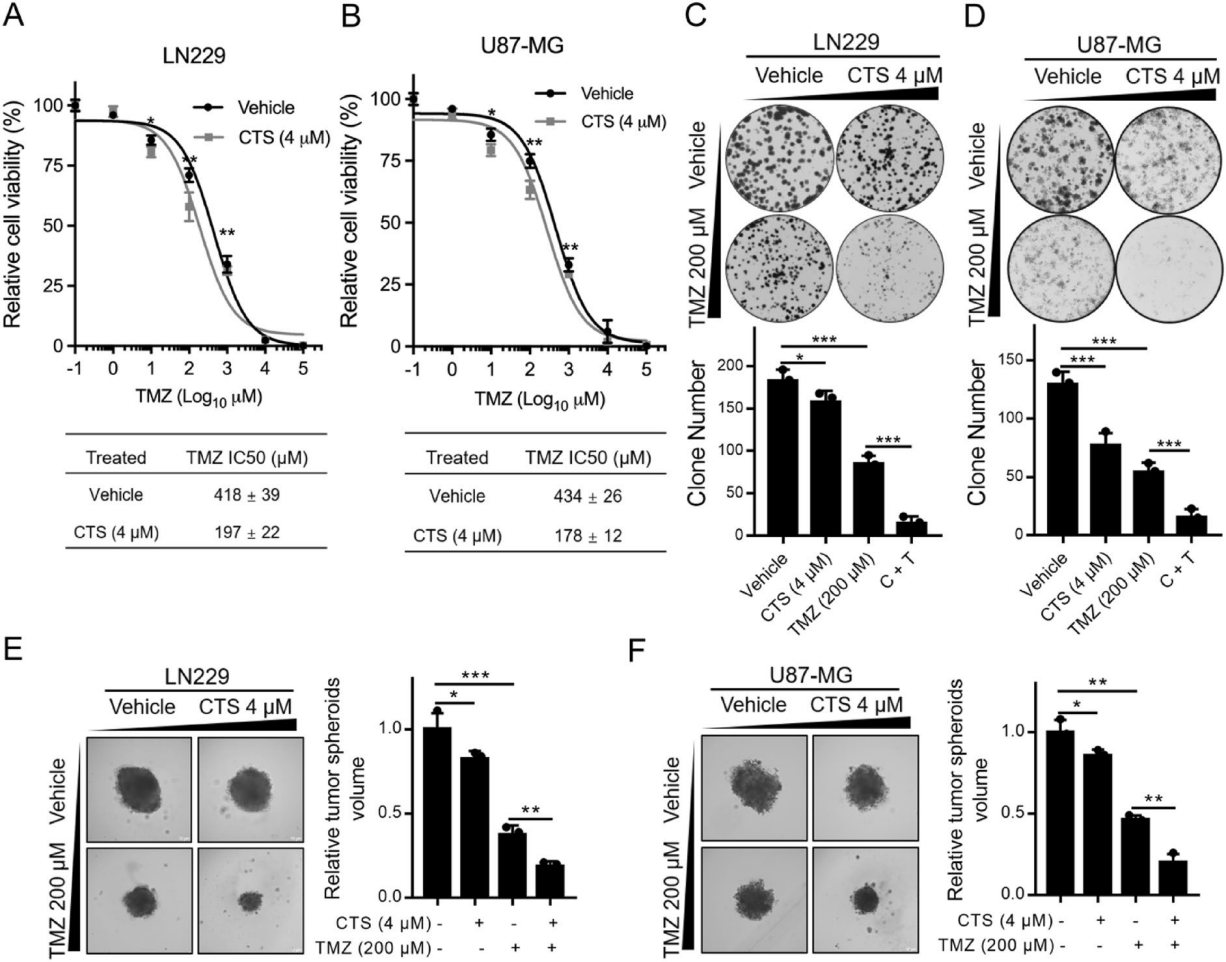
Low-dose CTS and TMZ combination treatment suppresses cell proliferation. (**A**,**B**) IC50 values of TMZ treatment for 72 h with or without CTS of LN229 and U87-MG GBM human cells. (**C**,**D**) Representative images (upper panels) and quantification (lower panels) of the colony formation assays of LN229 and U87-MG cells treated with the indicated concentrations of TMZ and CTS. (**E**,**F**) Representative images (left panels) and quantification (right panels) of 3D spheroids formation assays of LN229 and U87-MG cells treated with the indicated concentrations of TMZ and CTS (***P < 0.001, **P < 0.01, *P < 0.05).

To further confirm whether CTS combined with TMZ has a synergistic inhibitory effect on glioma cells, LN229 and U87-MG cells were treated with a series of TMZ concentrations, with or without CTS treatment, for 72 h, and the IC50 value of TMZ was assessed using a cell viability assay. As shown in Fig. 3A,B, compared with the control group, the IC50 value of TMZ decreased from 418 to 197 μM following the combination treatment of TMZ and CTS. Subsequently, we evaluated the long-term inhibitory effect of TMZ combined with CTS on GBM cells using colony and 3D spheroid formation assays. The combination of TMZ and CTS substantially suppressed colony formation in LN229 and U87-MG cells (Fig. 3C,D) and 3D spheroid formation (Fig. 3E,F) compared with that of either treatment alone.

### Combined treatment with CTS and TMZ synergistically induced DNA alkylation damage and apoptosis

**Figure 4:**
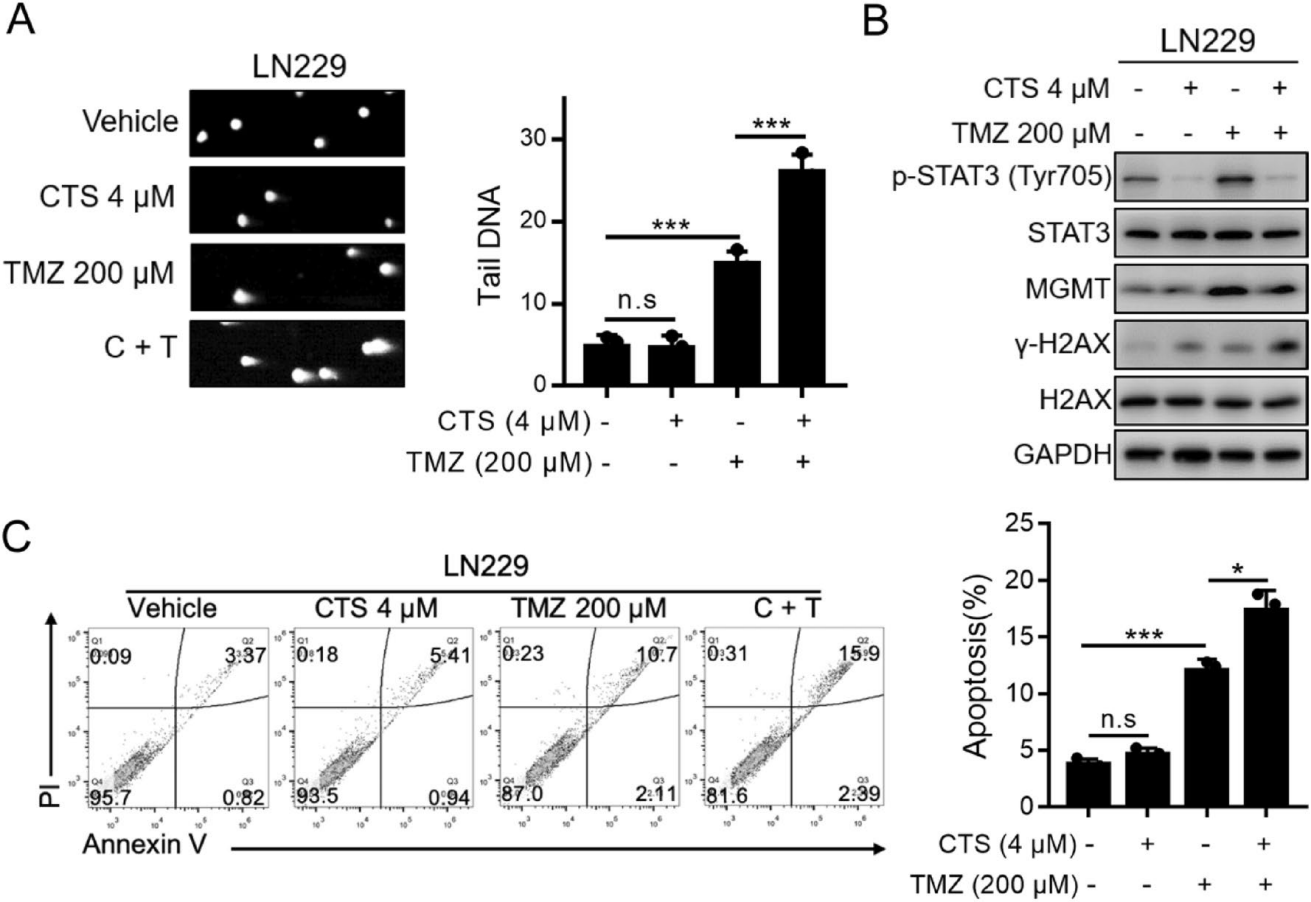
Combining CTS and TMZ treatments synergistically induce oxidative DNA damage and apoptosis. (**A**) Representative images (left panels) and quantification (right panels) of comet assays of LN229 human GBM cells treated with the indicated concentrations of TMZ and CTS. (**B**) The combination of CTS and TMZ treatments synergistically induced DNA damage, as confirmed by western blot analysis. (**C**) Representative images (left panels) and quantification (right panels) of apoptosis following the combination of CTS and TMZ treatments in LN229 cells (***P < 0.001, **P < 0.01, *P < 0.05).

Next, we determined the combined effect of TMZ and CTS treatment on GBM cells. We treated LN229 cells with vehicle, 4 μM CTS, 200 μM TMZ, or both CTS and TMZ for 72 h and detected the proportion of cells with DNA damage in each group. As shown in Fig. 4A, the significantly elongated tailings ratio of the combination treatment relative to either the control or single agent treatments suggests that more fragments of DNA damage were present after treatment with both CTS and TMZ. The inhibition of STAT3 activation (p-STAT3) reduces MGMT expression^30^. As the nucleosome histone H2AX is rapidly phosphorylated at Serine 139 (γ-H2AX) at the double-strand break (DSB) site, γ-H2AX formation is commonly used to quantify DSBs. To confirm whether the combination of CTS and TMZ enhances the toxic effect of these drugs on tumor cells, western blot analysis was performed to detect the expression of these proteins. As shown in Fig. 4B, p-STAT3 (Tyr705) expression was negligible with CTS treatment alone, whereas the expression of γ-H2AX was slightly increased. However, after TMZ treatment, MGMT protein expression was significantly upregulated. Meanwhile, compared with TMZ single-agent treatment, MGMT expression was significantly decreased, and γ-H2AX expression was significantly increased with the CTS and TMZ combined treatment. TMZ-induced DNA damage may be blocked by STAT3, which is inhibited by CTS during the activation of MGMT expression.

Finally, we observed the effect of combination therapy on apoptosis. As shown in Fig. 4C, apoptosis was significantly induced by CTS and TMZ combined treatment and compared with the TMZ single-agent. Therefore, CTS combined with TMZ induced a higher ratio of cell apoptosis.

### CTS reverses TMZ resistance by targeting STAT3 to suppress MGMT expression in TMZ-resistant cell models

**Figure 5:**
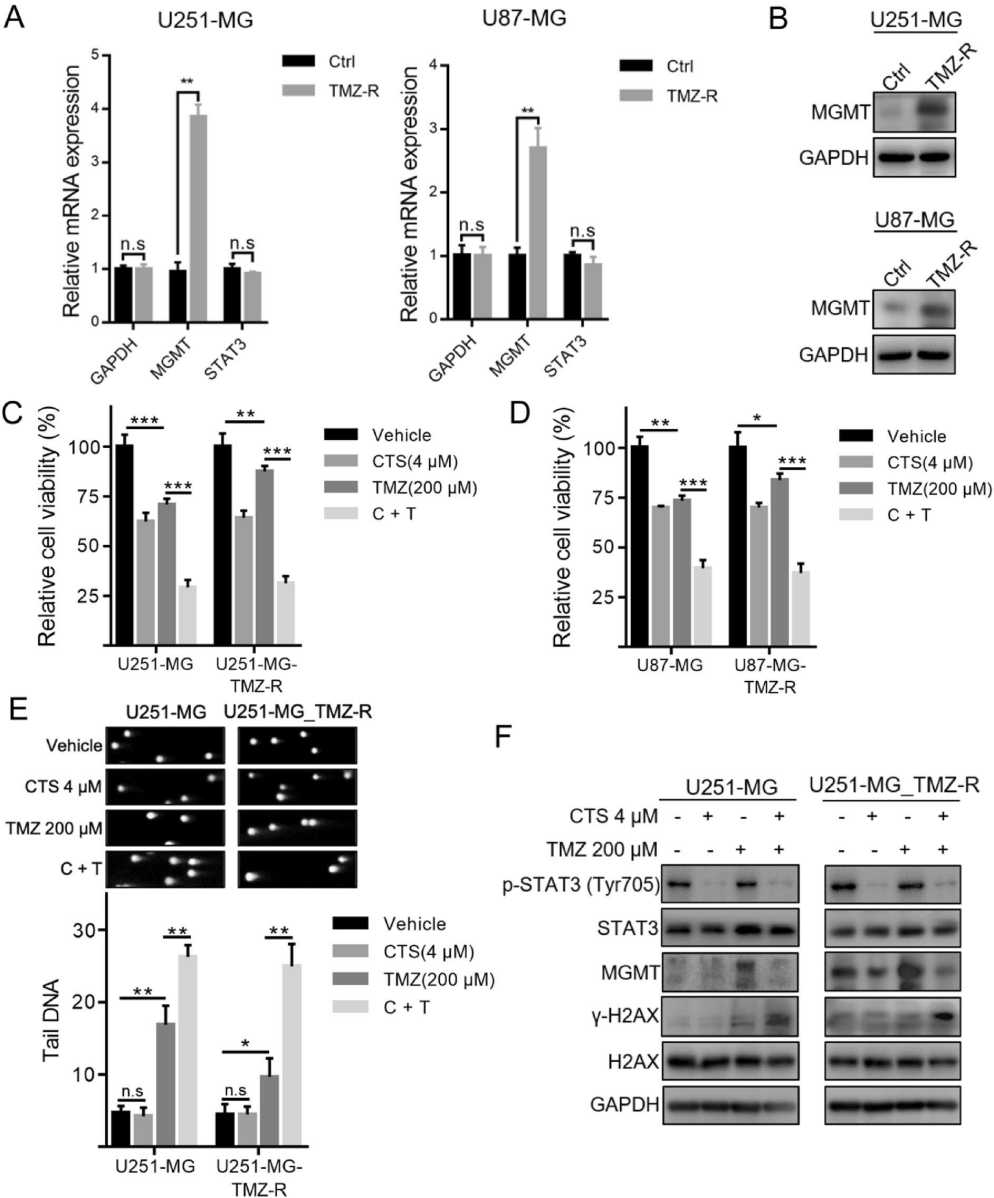
Combined treatment of CTS and TMZ reversed TMZ resistance of human GBM cells. (**A**) Validation of TMZ-resistant cell models by qRT-PCR. (**B**) Validation of TMZ-resistance cell models by western blot analysis. (**C**,**D**) Cell viability of each treatment with the indicated concentrations of TMZ and CTS in TMZ-resistant cell models. (**E**) Representative images (upper panels) and quantification (lower panels) of comet assays in TMZ-resistant cells treated with the indicated concentrations of TMZ and CTS. (**F**) The combination of CTS and TMZ treatments synergistically induced DNA damage and reversed TMZ resistance, as confirmed by western blot analysis (***P < 0.001, **P < 0.01, *P < 0.05).

To confirm the promotional effect of CTS on TMZ-induced cytotoxicity, we conducted further studies using TMZ-resistant (TMZ-R) cell models. As mentioned previously, TMZ-resistant cell models were obtained by low-dose stimulation, and qRT-PCR and western blot analyses demonstrated that *MGMT* mRNA and protein expression was significantly increased (Fig. 5A,B). Next, we compared the inhibitory effect of CTS combined with TMZ on the growth of wild-type and TMZ-resistant cells through cell viability experiments. We observed that CTS exhibited superior inhibition effect on both TMZ-resistant and wild-type cells. The combination of CTS and TMZ significantly enhanced the inhibiting effect of TMZ on TMZ-resistant cells, whereas the inhibition of TMZ on U251-MG_TMZ-R and U87-MG_TMZ-R cells dramatically reduced at the same concentration when compared to wild-type cells (Fig. 5C,D). Next, we observed the DNA damage effects of CTS combined with TMZ on GBM cells. As shown in Fig. 5E, CTS treatment alone had no effect on DNA damage in U251- or U87-TMZ-R cells. With 200 µM TMZ treatment, DNA damage in U251 cells increased significantly, whereas that in U251-TMZ-R cells did not. Finally, western blot analysis was used to detect the expression of γ-H2AX and MGMT in wild-type and TMZ-resistant cells treated with CTS and TMZ. As shown in Fig. 5F, MGMT background expression in U251-TMZ-R cells was relatively high compared with that in U251-MG cells. TMZ stimulation further increased the MGMT protein expression. Conversely, MGMT expression in TMZ-resistant cells treated with TMZ and CTS was inhibited, which promoted an increase in TMZ-induced DNA damage.

## Discussion

Human GBM is one of the most common, aggressive, and malignant brain tumors of the central nervous system in adults. As the first-line option, TMZ-based chemotherapy is a routine and crucial therapy for GBM, followed by standard surgical excision. However, glioma cells eventually metastasize and develop chemoresistance, which is an obstacle to the therapeutic efficacy of TMZ in GBM. Therefore, the molecular mechanisms underlying the suppression of TMZ resistance must be elucidated to identify and develop potential novel molecular targets for GBM therapy. In the present study, we demonstrated that CTS treatment suppressed STAT3 activity and arrested GBM cell proliferation in a dose- and time-dependent manner. Given that STAT3 is a crucial target for a number of biological processes, inhibition of STAT3 activity mediated by CTS does not completely prevent cell proliferation. We investigated the possibility of enhancing the anti-glioma efficacy of TMZ by combination therapy, considering the significance of TMZ in glioma chemotherapy and the fact that CTS reduces tumor cell proliferation. Based on the synergy score evaluation, we identified the optimal concentrations of the anti-glioma drugs CTS and TMZ. In this study, we demonstrated for the first time that CTS has a combined effect with TMZ, which effectively inhibits colony formation and tumor spheroid proliferation. We further confirmed that CTS, in combination with TMZ, had an enhanced therapeutic effect on LN229 and U87-MG GBM cells compared with that of CTS or TMZ treatment alone. In addition, CTS combined with TMZ effectively reduced the required dosage of TMZ. MGMT is a DNA repair protein that widely exists in organisms from bacteria to mammals, and can repair DNA alkylation damage caused by TMZ, leading to TMZ resistance in GBM cells. The heavy dependence of cancer cells on MGMT-mediated DNA repair makes the regulation of MGMT expression an attractive target for cancer therapy. In this study, DNA fragmentation was analyzed using a comet assay. The significantly elongated tailings ratio of combination treatment relative to control or single-agent treatment suggests an increased abundance of DNA-damaged fragments following treatment with both TMZ and CTS. Furthermore, the accumulation of DNA damage caused by the inhibition of MGMT expression led to the apoptosis of tumor cells. We also observed that low-dose CTS treatment resulted in a better inhibition efficiency of TMZ-R cells than in the control group. However, TMZ-R cells reversed their insensitivity to TMZ when co-treated with CTS. Our work validates the mechanism by which CTS reverses TMZ resistance and induces cell DNA damage in TMZ-R GBM cells by inhibiting STAT3 activation to decrease MGMT expression and enhance TMZ-induced MGMT-dependent DNA damage.

In conclusion, we found that CTS exhibited a time- and dose-dependent inhibition of glioma cell proliferation, and that the synergistic interaction between CTS and TMZ increased the curative effects of TMZ. The primary mechanism could be that CTS suppresses MGMT expression by inhibiting STAT3 activation, thus enhancing apoptosis induced on by TMZ alkylation. Although our findings point to the possible advantages of CTS and TMZ combination therapy, the comprehensive molecular modification of the STAT3 signaling pathway by CTS must be investigated to explore its application in other malignant diseases.

## Materials and methods

### Reagent preparation and storage

CTS and TMZ were purchased from MedChemExpress (MCE, New Jersey, USA). CTS was dissolved in 100% DMSO to prepare a stock solution at a concentration of 10 mM; TMZ was dissolved in 100% DMSO to prepare a stock solution at a concentration of 100 mM. Both were equipped and wrapped in a brown centrifugal tube for protection against light and stored at − 20 °C.

### Cell line and cell culture

The two typical human GBM cell lines LN229 and U87-MG were obtained from the Institute of Biochemistry and Cell Biology, Chinese Academy of Science. Cells were maintained in Dulbecco’s Modified Eagle Medium (glucose 4.5 g/L; Gibco, NY, USA) with 10% Fetal Bovine Serum (Gibco, NY, USA) and penicillin (100 U/mL)-streptomycin (100 μg/mL) in a humidified incubator at 37 °C with 5% CO_2_ and authenticated using a short tandem repeat (STR) assay (Genetic Testing Biotechnology, Jiangsu, China).

### Cell viability assay

Cell viability was determined using Cell Counting Kit 8 (CCK-8) from Beyotime (Beyotime, Shanghai, China), stored at 4 °C. Following being seeded at a population density of 2 × 10^3^ in 96-well plates for 24 h, the cells were treated for 72 h with either CTS, TMZ, or CTS + TMZ. Then, the cells were treated for 1 h at 37 °C with 10 µL of CCK-8. Utilizing a 450 nm absorbance measurement, the quantity of viable cells was ascertained.

### Establishment of TMZ‐resistant GBM cell line

The U251-MG and U87-MG TMZ-resistant cell line was developed by using the TMZ concentration gradient progressive methods as previous described^33^. Briefly, 1 × 10^5^/mL of U251-MG or U87-MG cells were inoculated in TMZ-free culture medium for 24 h until they got to the logarithmic phase. Then the culture medium was replaced with that containing low concentration (50 µM) TMZ for 72 h. Then the culture medium containing drugs and dead cells was discarded. Cells were collected and re-inoculated in TMZ-free culture medium to get recovered before the next TMZ treatment. After the cells got adjusted to the present TMZ treatment, the concentration of TMZ was increased in turn until the cells survived well and developed resistance to the 400 µM TMZ treatment.

### Quantitative real-time polymerase chain reaction (qRT-PCR)

Cellular RNA was isolated using TRIzol reagent (Invitrogen, CA, USA) and reverse transcribed to complementary DNA (cDNA) using a cDNA Synthesis Kit (Vazyme, Nanjing, China). For QPCR, we used *GAPDH* gene Forward primer 5′-GTCTCCTCTGACTTCAACAGCG-3′, *GAPDH* gene reverse primer 5′-ACCACCCTGTTGCTGTAGCCAA-3′; *MGMT* gene forward primer 5′-ACCGTTTGCGACTTGGTACTT-3′, *MGMT* gene reverse primer 5′-GGAGCTTTATTTCGTGCAGACC-3′; *STAT3* gene forward primer 5′-CCTGCTAAAATCAGGGGTCC-3′, *STAT3* gene reverse primer 5′-GCTTCTCCCCCTCGGCT-3′. The SYBR Green RT-PCR Kit (Vazyme, Nanjing, China) was used for qRT-PCR and the thermal cycle parameters used were: 95 °C for 30 s, 40 cycles of 95 °C for 10 s, and 60 °C for 30 s. Each reaction was repeated in triplicate following the manufacturer’s protocol. mRNA levels in each well were normalized to that of glyceraldehyde-3-phosphate dehydrogenase (GAPDH) using the 2^−ΔΔCt^ method (Supplementary Figures).

### Colony formation assay and 3D spheroid formation assay

For colony formation assay, cells were treated with CTS, TMZ, or CTS + TMZ for 72 h following their seeding in 12-well plates at a population density of 200 per well for 4 days. Colonies were fixed with methanol and stained with crystal violet, following the manufacturer’s protocol. The plating efficiency was determined by counting the number of colonies and was calculated as follows:

$$({\text{Number of colonies formed/Number of cells inoculated}}) \times 100\%.$$

For the 3D spheroid formation assay, 200 cells per well were seeded in ultra-low-binding 96-well plates with a round bottom (Costar, MA, USA). After nine days of treatment with either CTS, TMZ, or CTS + TMZ, digital images of the spheroids were captured using a phase-contrast microscope (Leica, Weztlar, Germany). The volumes of the 3D spheroids were calculated using the formula “(V = 0.5 × Length × Width^2^)” based on the major and minor axial lengths (Length: major axial length; Width: minor axial length).

### Comet assay

For comet assay, cells were treated with CTS, TMZ, or CTS + TMZ for 72 h following their seeding in 6-well plates at a population density of 2 × 10^5^ for 24 h. Then, cells were harvested, washed, and resuspended in phosphate-buffered saline (PBS) at a concentration of 1 × 10^6^ cells/m. Subsequently, low melting agarose was dissolved in the cell suspension to generate a 0.75% agarose mixture. 100 μL normal melting agarose and 100 μL cell-agarose mixtures were separately added to a fully frosted slide at 10 min intervals to form two layers and were incubated at 4 °C for 30 min. After removing the coverslip, the slide was incubated in lysis buffer (10 mM Tris, pH 10.0, 2.5 M NaCl, 0.1 M EDTA2Na, 10% DMSO, and 1% Triton X-100) at 4 °C overnight. The slide was subjected to electrophoresis in Mini Horizontal Cells (Bio-Rad, CA, USA) filled with ice-cold alkaline electrophoresis buffer (0.3 M NaOH, 1 mM EDTA) and ran at 300 mA and 25 V for 30 min. After soaking in neutralization buffer (0.4 M Tris–HCl, pH 7.5) for 5 min, the slide was counter-stained with 5 mg/mL of the nuclear stain DAPI. Images were acquired using a Leica Thunder microscope and analyzed using the Comet Score software (CASP, CASP-Lab).

### Flow cytometry

For flow cytometry assay, cells were treated with CTS, TMZ, or CTS + TMZ for 72 h following their seeding in 6-well plates at a population density of 2 × 10^5^ for 24 h. Then, cells were harvested and washed with PBS. A total of 2 × 10^5^ cells were added to 100 μL binding buffer to create a cell suspension, followed by the addition of 5 μL Annexin V and 10 μL propidium iodide (PI) in sequence. Once mixed, this preparation was incubated for 25 min at 4 °C. After the further addition of 200 μL of binding buffer, flow cytometry was performed, and cell apoptosis was determined. The extent of apoptosis was measured using an Annexin V-FITC apoptosis detection kit (Beyotime, Shanghai, China) and analyzed using flow cytometry software (Beckman, USA).

### Western blotting analysis

Following being seeded at a population density of 3 × 10^5^ in 6-well plates for 24 h, the cells were treated for 72 h with either CTS, TMZ, or CTS + TMZ. Cells were lysed and homogenized in radioimmunoprecipitation assay (RIPA) buffer, and protein concentrations were determined using a BCA Protein Assay Kit (Thermo Fisher, CA, USA). Protein samples were separated using 10% sodium dodecyl sulfate–polyacrylamide gels and transferred to polyvinylidene fluoride membranes (Merck Millipore, MA, USA). The membranes were blocked with 3% bovine serum albumin for 1 h. The blots were incubated with specific primary antibodies overnight at 4 °C and then incubated with corresponding horseradish peroxidase-conjugated secondary antibodies for 1 h. The primary antibodies used were anti-phospho-STAT3 (Y705) (1:5000; catalog no. 76315), anti-STAT3 (1:2000; catalog no. 68153), anti-MGMT (1:2000; catalog no. 39253), anti-H2AX (1:5000; catalog no. 124781), anti-γH2AX (1:5000; catalog no. 81299) (Abcam, MA, USA), and anti-GAPDH (1:4000; catalog no. 10494-I-AP) (Proteintech, Chicago, USA). Finally, the protein bands were visualized using an ECL kit (Beyotime, Shanghai, China), and the density of the immunoreactive bands was analyzed using Imager software (Tanon, Shanghai, China).

### Statistical analysis

Statistical analysis was performed with Graph-Pad Prism7 (version 7.0; GraphPad Software, Inc., San Diego, CA, USA). The unpaired two-group comparison and multiple comparisons were made with the Student’s t-test or one-way ANOVA, respectively. All data are presented as the mean ± standard deviation (mean ± SD). Differences between means were considered statistically significant when *P* < 0.05 (*P < 0.05, **P < 0.01, ***P < 0.001). The synergistic effect analysis of the two drug combinations was performed using *Combinefit 2.02* software (Combinefit, Inc., San Diego, CA, USA).

### Supplementary Information

Supplementary Figures.

## Data Availability

The data presented in this study are available in the article.
