# Supplementary figures and images for: Synergistic effect of cryptotanshinone and temozolomide treatment against human glioblastoma cells
Source: Sci Rep. 2023 Dec 9;13:21835. doi: 10.1038/s41598-023-48777-z (PMC10710453; doi:10.1038/s41598-023-48777-z)

**Supplementary figures:**


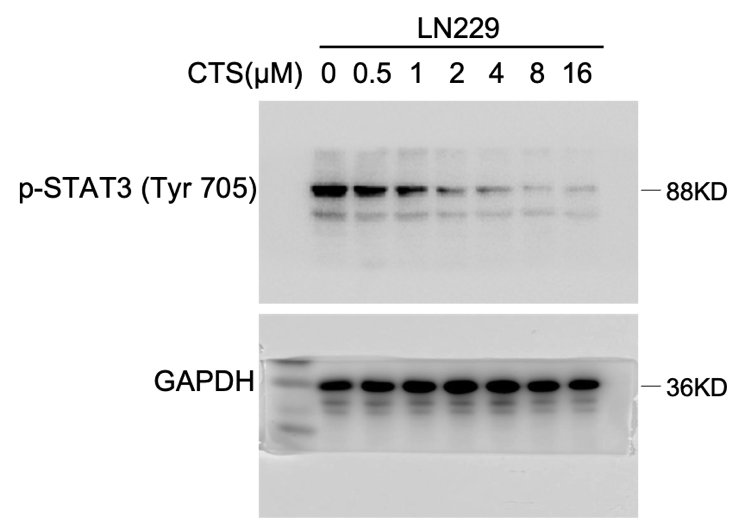
Figure 1D:


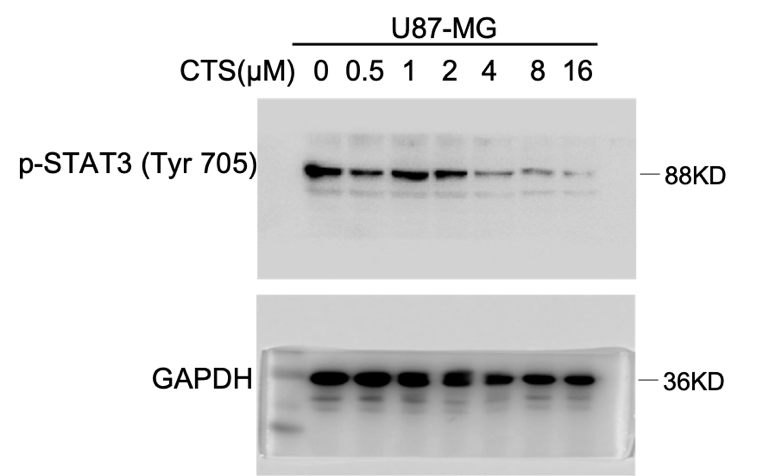
Figure 1E:


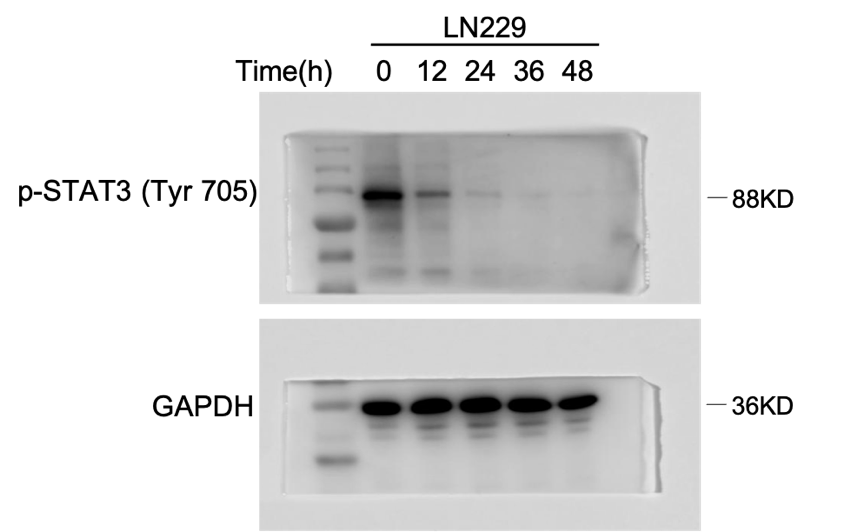
Figure 1H:


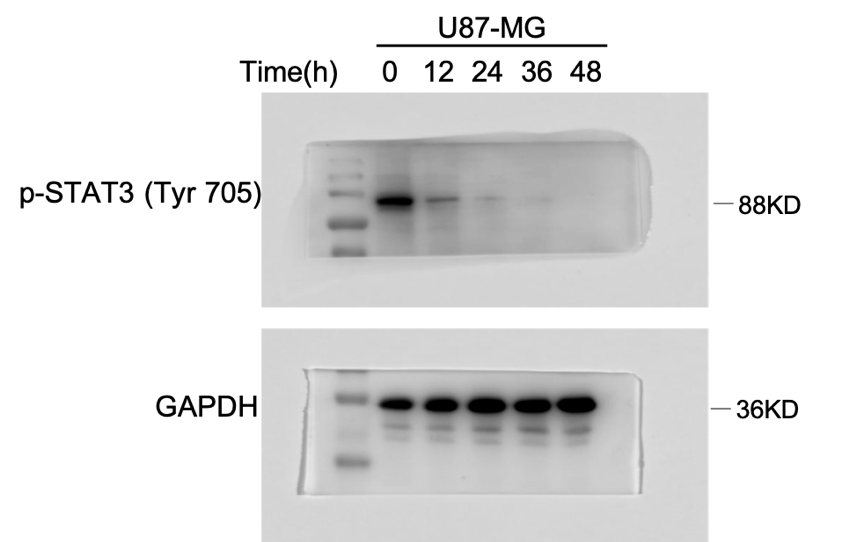
Figure 1I:


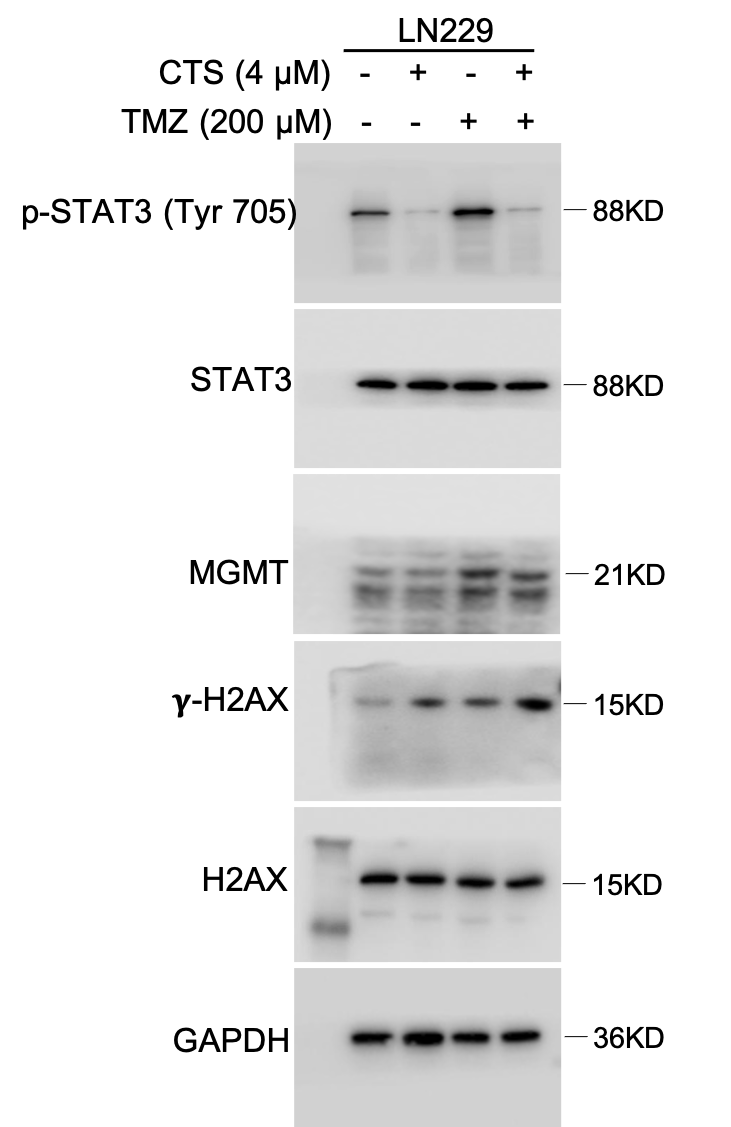
Figure 4B:


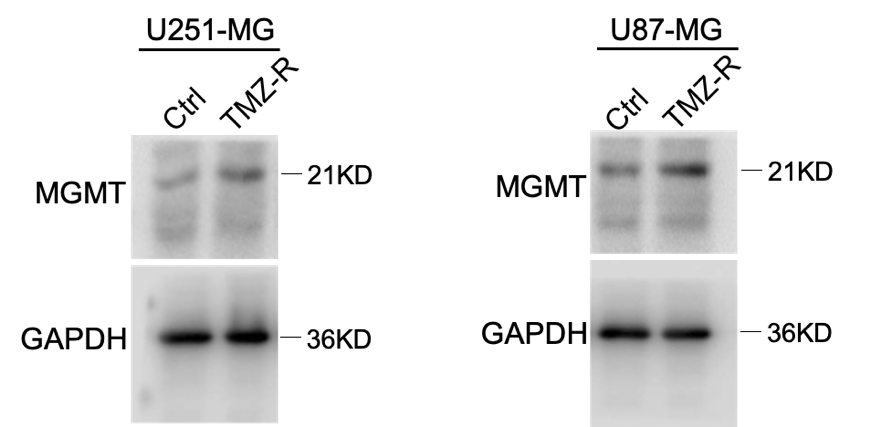
Figure 5B:


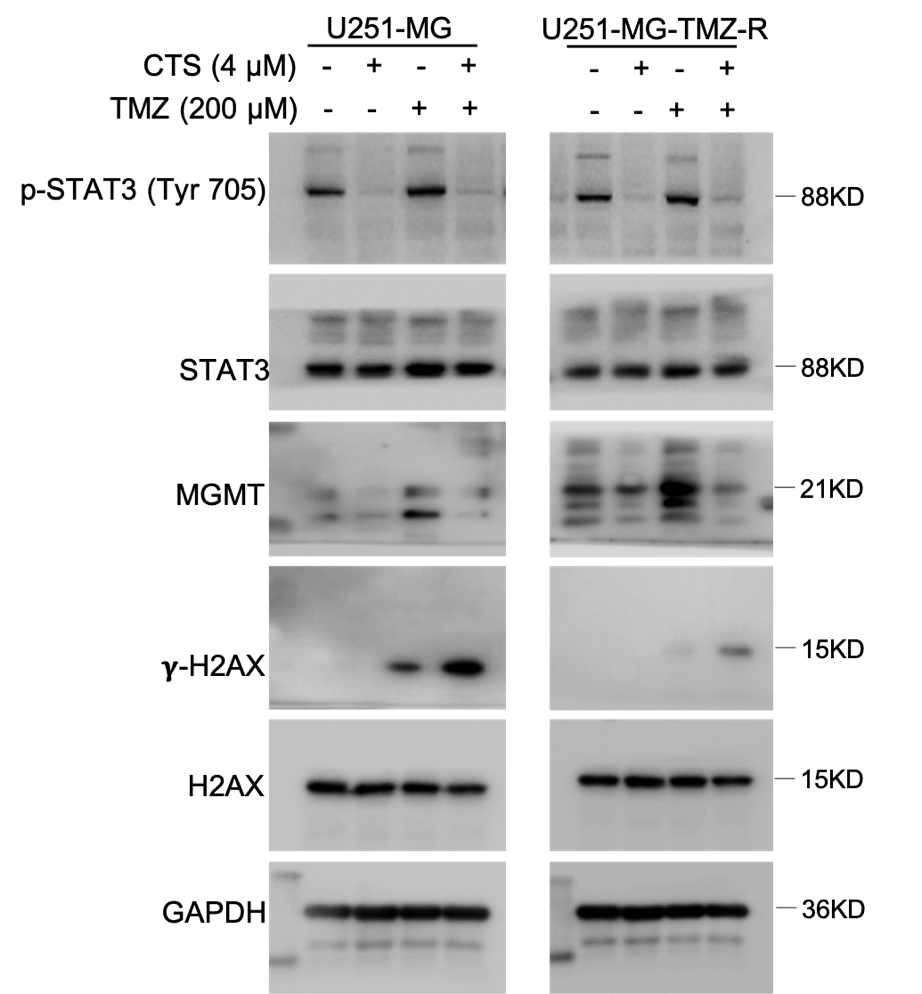
Figure 5F:

Supplement: Supplementary file 1 — Supplementary Figures. [file 41598_2023_48777_MOESM1_ESM.docx]
